# P-1836. County by county estimation of HIV, Hepatitis C Virus, and deaths averted caused by Overdose Prevention Health Centers in the United States from January 2020-June 2024

**DOI:** 10.1093/ofid/ofaf695.2005

**Published:** 2026-01-11

**Authors:** Mike Sportiello, Rohith Palli

**Affiliations:** Emory University, Atlanta, GA; University of Rochester, Rochester, New York

## Abstract

**Background:**

Overdose prevention health centers (OPHCs), also known safe injection facilities, are one public health strategy wherein people use drugs in an environment monitored by healthcare professionals with access to safer drug use education, sterile supplies, and referral infrastructure to access higher levels of medical and behavioral health resources. Though still illegal under federal law throughout the United States, many legal or quasi-legal (including some in the United States) have operated since the 1980s. While individual estimates for individual counties have been done, no country-wide estimate exists for possible benefits of OPHCs. Herein, we estimate the impact of OPHC construction within each county on new Hepatitis C Virus (HCV) and HIV transmissions, as well as the effect on overdose deaths.

**Figure:**
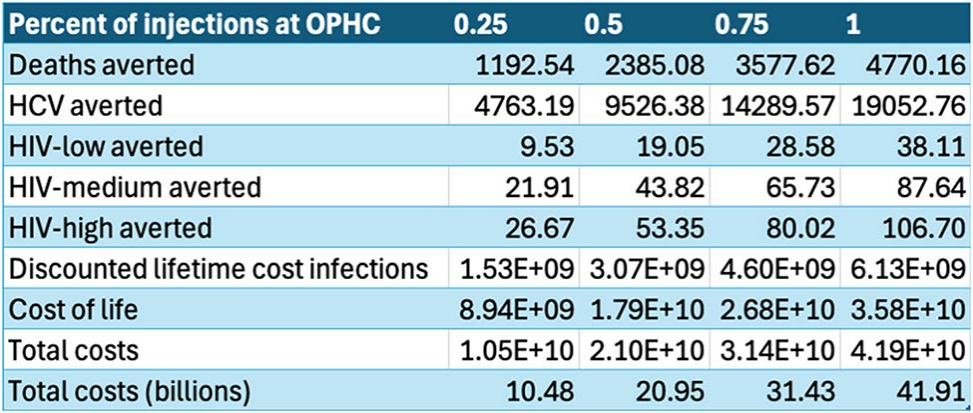
Results table Infections averted, deaths averted, and cost savings from OPHC construction by injection frequency at Overdose Prevention Health Center.

**Figure:**
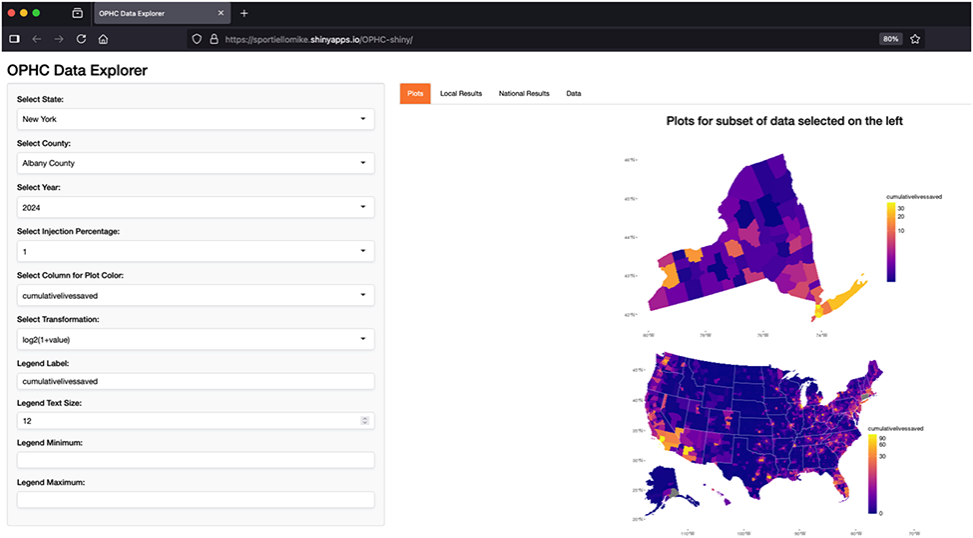
OPHC interactive data explorer screenshot Publicly available, open-source web interactive for users to explore data and modulate models within set constraints.

**Methods:**

Publicly available county-level data including population, age distribution, and overdose deaths were downloaded from the CDC and US Census Bureau, and county-level estimates of the numbers of people who inject drugs were made. OPHC uptake rates, as a function of frequency of injections occurring at an OPHC, were estimated to fall between 0 and 1% of all injections. A publicly available, free and open-source web application (https://sportiellomike.shinyapps.io/OPHC-shiny/) was created to visualize the data and allow the user to alter variables within our model, within appropriate constraints. Estimates for HIV and HCV transmissions averted, deaths averted, deferred costs of treating said infections, and more are available to the user.

**Results:**

Using default settings on our model and assuming 1/400 injections occurred at an OPHC between January 2020 and June 2024, 4,763 HCV cases, 27 HIV cases, and 1,193 deaths would have been averted translating to over $1.5 billion in discounted infectious costs.

**Conclusion:**

OPHCs remain a nearly untapped source of cost-effective harm reduction available to avert infections and deaths within the United States. The greatest limitation of this work include imprecise estimation of uptake of OPHC use by community members and secondary impact of OPHC benefits including referral to addiction treatment and supplying of overdose reversal agents and sterile supplies for use outside of the OPHC.

**Disclosures:**

All Authors: No reported disclosures

